# Perceptive Body Image Distortion in Adolescent Anorexia Nervosa: Changes After Treatment

**DOI:** 10.3389/fpsyt.2019.00748

**Published:** 2019-10-15

**Authors:** Anke W. Dalhoff, Hugo Romero Frausto, Georg Romer, Ida Wessing

**Affiliations:** ^1^Department of Child and Adolescent Psychiatry, University Hospital, Münster, Germany; ^2^Institute for Biomagnetism and Biosignalanalysis, University Hospital, Münster, Germany

**Keywords:** anorexia nervosa, body image distortion, body size estimation, adolescents, inpatient treatment

## Abstract

One key symptom of anorexia nervosa (AN) is body image distortion (BID). For example, AN patients who are asked to perform body size estimation tasks tend to overestimate their body size; this is thought to indicate a distortion of the perceptive component of body image. Although BID is an important treatment objective, only few treatment approaches explicitly target body image, and even fewer target the perceptive component. Moreover, very little is known about how patients’ perceptive body image changes after treatment and related weight gain. Consequently, we investigated changes of the perceptive BID in adolescent AN patients at the beginning (T1) and the end (T2) of inpatient treatment using a body size estimation task. A total of 38 AN patients performed the test for Body Image Distortion in Children and Adolescents (BID-CA) within the first 2 weeks of inpatient treatment and at the end of treatment. The results were compared to 48 healthy control (HC) participants performing the same task once. At T1, AN patients overestimated their body size more than HC, i.e., a total overestimation of 33% in AN patients *vs*. 11% in HC. At T2, AN patients overestimated their arm size to the same degree that they did at TI, but overestimations for the thigh and waist were reduced, and their overestimations for the waist no longer differed from the HC group. Thus, after treatment, AN patients were partly able to more realistically estimate their body size. Several factors may have influenced the observed changes in body size estimation, including task repetition, deliberate adjustment, growing into their preexisting perceptive body image through weight gain, as well as targeted and non-specific psychotherapeutic treatments. In conclusion, the perceptive BID in adolescent AN patients is persistent but also modifiable. Although diverse factors presumably play a role in changing BID, these findings suggest that AN patients may benefit from targeted treatment of BID.

## Introduction

Anorexia nervosa (AN) is an eating disorder associated with restrictive food intake and severe underweight (1). One key symptom of AN that often motivates this detrimental dietary behavior is BID, including negative feelings and appraisals toward the body (cognitive–affective component) and body size overestimation (perceptive component). BID may be highly persistent and is known to be prognostic for both the initial manifestation (2, 3) and the short- and long-term outcomes of the disorder (4–6).

The cognitive–affective component of the body image is typically measured by self-report questionnaires or interviews. It comprises different aspects that patients are usually consciously aware of and well able to describe, including, e.g., body dissatisfaction, overvaluation of shape or weight, preoccupation with shape or weight, and fear of weight gain (7). Despite diverse methods of measurement and ongoing debates on the relative importance of different aspects, there is a consensus that the cognitive–affective component of body image is strongly affected and negatively distorted in AN (7–9); thus, the cognitive–affective aspects of body image are an important treatment objective (10). Indeed, targeted treatment leads to clear improvements in body weight and overall eating disorder psychopathology (e.g., eating concern and weight concern). Interestingly though, the improvements in the cognitive–affective aspects that are particularly related to the perception of one’s own body (shape concern, feeling fat) seem to be less pronounced (5, 11).

The perceptive component of the body image is related to AN patients’ tendencies to perceive their own body as bigger than it actually is. This component is less conscious in everyday life, but it can be measured by visual and metric body size estimation tasks (12, 13). In visual body size estimation tasks, images of one’s own body or stylized bodies with different sizes are presented, and participants have to select or configure the picture so that it best matches their own body. In metric body size estimation tasks, AN patients first estimate the size of several body parts, and, second, these body parts are measured, e.g., using a rope or caliper. Both methods typically include the calculation of index values relating the estimated and real body sizes to indicate the amount of overestimation. A recent review and a meta-analysis of studies using such tasks (12, 13) conclude that there is clear evidence of body size overestimation in AN. Moreover, in adolescent AN patients, less body size overestimation predicted a better long-term outcome (4). This strongly suggests that the perceptive component of BID should also be a treatment objective. However, so far, only few treatment approaches directly target the perceptive component of BID.

One such treatment approach is body exposure using a mirror or video feedback. This technique targets both AN patients’ tendencies to have a distorted visual perception and a negative cognitive evaluation of their body (14, 15). Indeed, this technique has been shown to reduce visual body size overestimation (16). However, it is unclear whether this visual overestimation in AN patients is due to an impaired visual perception *per se*, as several studies have reported that AN patients have intact vision (17) and visuospatial processing (18). An alternative explanation may be given based on the consideration by Molbert et al. (13) that visual size estimation tasks assess explicit (but not implicit) representations of the body. It may be argued that overestimation in visual body size estimation tasks, as well as changes in visual overestimation after treatment, are driven by explicit cognitive–affective aspects to a large part.

Another approach is hoop training (19), in which patients learn to choose and move through a hoop that best fits their body size. This technique addresses multisensory body perception, including visual, tactile, and proprioceptive perceptions. This approach works to incorporate recent findings that indicate AN involves not only visual distortion but also nonvisual multisensory impairments, including tactile and proprioceptive perception and multisensory integration (20–22). In their pilot study, Keizer et al. (19) found that hoop training specifically improved results in the hoop task, while both treatments as usual and additional hoop trainings led to improvements in visual and tactile estimation tasks. As the sample size was very small, with only 14 AN patients completing the hoop training, this approach’s potential to improve multisensory body perception requires further investigation. Nonetheless, although no statistics were calculated, the results suggest that, across treatment groups, perceptive BID was persistent but less pronounced after treatment. This is further supported by similar findings in AN patients who completed eating disorder treatment (23). While treated AN patients did not differ from healthy control (HC) participants with respect to their cognitive–affective body image, their perceptive BID was persistent but less pronounced after treatment.

Overall, the treatment approaches mentioned above have aimed to address the perceptive component of body image, namely, the visual aspect (*via* the body exposure approach) or multisensory aspects (*via* hoop training) of body perception. Further, findings from these studies have shown that visual body exposure indeed improved BID in a visual size estimation task (16), and hoop training improved performance in the respective hoop task (19). Nevertheless, even though BID is a key symptom and highly relevant treatment objective in patients with AN, so far, only very few studies have investigated changes of the perceptive BID in AN, both after targeted or non-targeted treatments. Hence, little is known about how AN treatment and associated weight gain modifies the perceptive BID. Moreover, to the best of our knowledge, no studies have used a metric body size estimation task before and after treatments. Metric body size estimation tasks are considered to assess more implicit perceptive body representations (13) and are, therefore, particularly suited to capture aspects of the body image that are less conscious and, hence, perhaps difficult to change.

The aim of the present study was, therefore, to investigate changes of perceptive BID in adolescent patients with AN. To this end, AN patients completed a metric body size estimation task (Test for Body Image Distortion in Children and Adolescents; BID-CA; (24) within the first 2 weeks of inpatient treatment and at the end of treatment. Treatment comprised a phased multimodal treatment plan that included a disorder-specific body-psychotherapeutic treatment approach. We expected to replicate previous findings of body size overestimation in adolescent AN patients compared to HC participants (24–26). Based on previous findings in AN patients who received treatment targeting perceptive BID (16, 19) and in AN patients who had completed eating disorder treatment (23), we expected body size overestimation in AN patients to persist after treatment but to be less pronounced than before treatment.

## Materials and Methods

### Participants

This study included female participants aged 12 to 19 years. The study protocol, including partly retrospective data collection, was approved by the ethics committee of the Medical Association of Westphalian-Lippe and the University of Münster. Participants in the HC group were recruited in secondary schools in Münster, Germany. They had to have a BMI between the 10^th^ and 90^th^ age percentiles and no current or prior mental illness according to structured clinical interviews, i.e., EDE-I (27) and K-DIPS (Unnewehr, Schneider, & Margraf). Patients with AN were recruited in a specialized ward for eating disorders at the Department of Child and Adolescent Psychiatry, University Hospital Münster, Germany. Patients had to have a primary diagnosis of AN or atypical AN (Eating Disorder Not Otherwise Specified; EDNOS). All comorbid diagnoses were included, except for neurodevelopmental, schizophrenia spectrum, or bipolar disorders. Diagnoses were based on clinical evaluation according to the criteria in the ICD 10. The fulfillment of the criteria in the DSM 5 was confirmed retrospectively based on the patient records and, if available, structured clinical interviews.

### Inpatient Treatment

All AN patients were admitted to a specialized ward for eating disorders at the Department of Child and Adolescent Psychiatry, University Hospital Münster, Germany, and treated with a phased multimodal treatment plan. Of special importance for the results of this study, all AN patients were treated several times a week with a disorder-specific body-psychotherapeutic approach based on the Concentrative Movement Therapy (CMT; www.dakbt.de). CMT is rooted in the psychodynamic approach and operates through the stimulation of sensory motor body experience and non-verbal and verbal symbolizations. The disorder-specific body-psychotherapeutic approach used here particularly focuses on the needs of AN patients. In a bottom-up fashion, therapeutic work starts with promoting the experience of one’s own body, whereby patients perform practical exercises that encourage conscious tactile, proprioceptive, and interoceptive perception (perceptive component of body image). Then, treatment goes on to integrate these perceptions with mental representations of the body and the self (cognitive–affective component of body image), and finally, AN patients are encouraged to exchange their bodily experiences with others and to solve related emotional conflicts in interpersonal relationships, e.g., in the patient group and in parent–child interaction units (interpersonal component).

### Procedure and Materials

#### Procedure

The data analyzed in this study were collected either as part of a larger study protocol that will not be reported here (all HC subjects and 19 AN patients) or retrospectively and anonymously from patient records (19 AN patients). All participants of the larger study received written and oral study information, and the participants and their parents gave their written informed consent. At the first date of the larger study, HC participants were weighed (in underpants only) and measured (barefoot, straight posture with heels, back, head, and palms at the wall) by an investigator, in the same way that AN patients are weighed and measured by nursing staff in the ward, and completed the structured clinical interviews, the body size estimation task (BID-CA), and psychopathology questionnaires (see below). For all AN patients, data were initially collected as part of the clinical routine and were then extracted from the patient records. However, only later were structured clinical interviews included in this routine, so these interviews were not always available (see *Results*). In all AN patients, weight, height, and BID-CA were collected at two time points: first, within the first 2 weeks of inpatient treatment (T1) and, second, after treatment (T2). To test patients after the largest possible weight gain, the T2 measurement was conducted at the end of treatment in our hospital, which could be at the end of inpatient treatment or during a following day patient or outpatient treatment. The target weight was defined as a BMI at the 25^th^ age percentile. This was not always achieved, and AN patients were included independent of their final weight status.

#### Questionnaire Data

Psychopathology was assessed by self-report questionnaires. Eating disorder symptoms were captured using the Eating Disorder Inventory for Children (EDI-C; German version) (28), and body dissatisfaction was assessed with the Body Shape Questionnaire (BSQ; German version) (29). Furthermore, symptoms of depression were captured with the Beck Depression Inventory 2 (BDI-II; German version) (30), and symptoms of anxiety with the Screen for Child Anxiety Related Disorders (SCARED-D; German version) (30).

#### Test for Body Image Distortion in Children and Adolescents (BID-CA)

The BID-CA (24) was used to assesses the perceptive component of BID. The test was conducted by the same person (body psychotherapist) in all AN patients, while HC participants were partly measured by a different trained investigator. Before the test started, participants were instructed to tune into the task by becoming aware of their own body and its form and were encouraged to palpate their body if desired to promote access to the implicit perceptive body image. Participants were then asked to estimate the size of their upper arm at the level of their armpit, their thigh at the level of their crotch, and their waist at the level of their belly button using a string. The correct positions were indicated by the investigator at her own body. The string was placed on a table, and the participants were asked to form a circle (with one end of the string connecting to the rest of the string) that equaled the size of their body parts. The length of the string needed to form the circle was determined using a tape measure (as the rope was 0.9 cm thick, the length was measured form the point where the inner part of the end of the string touched the rest of the string). Next, the actual circumference was measured using the string, and as before the length of the string that was needed to make a circle around the body part was determined using the tape measure. Three BID-Indices (arm, thigh, waist) were calculated by dividing the patient’s estimated circumference by the actual circumference (in cm) and multiplying the result by 100. As a result, BID-Indices with a value of 100 reflect a perfect estimation, while deviations above or below 100 reflect the percentage of over- or underestimation, respectively.

### Statistical Analyses

Statistical analyses were conducted using IBM SPSS Statistics 25. To test for group differences between AN patients and HC regarding age, body height, BMI, and psychopathology, pairwise group comparisons for age in month, body height in meters, BMI, and the global scores of all questionnaires (EDI-C, BSQ, BDI-2, SCARED-D) were conducted by independent *t*-tests. To test for group differences between AN patients and HC regarding BID-Indices at T1 and T2, respectively, two separate mixed analyses of variance (ANOVAs) were calculated with the between-subject factor being the group (AN_T1, HC, and AN_T2, HC) and the within-subject factor being the body part (arm, thigh, waist). *Post hoc* pairwise group comparisons for the BID-Indices of each body part were conducted by independent *t*-tests. As an additional statistical control for the fact that BID-Indices generally tend to increase with decreasing body size (31), one-way ANOVAs were calculated for each time point and each body part separately, with the between-subject factor being the group (AN, HC) and the measured body size being the covariate. Next, to test for changes in BID-Indices in the AN group from T1 to T2, a repeated measures ANOVA was calculated with the within-subject factors of time (T1, T2) and body part (arm, thigh, waist). *Post hoc* comparisons of T1 *vs*. T2 for the BID-Indices of each body part were conducted by dependent *t*-tests. Moreover, to investigate what drove possible BID-Index changes, the percentage change of the measured and estimated circumferences was calculated, correcting for overall size differences between body parts. Next, a repeated measures ANOVA with the factors of method (measured, estimated) and body part (arm, thigh, waist) were calculated, as were respective dependent *post hoc t*-tests. All statistical tests were corrected in the event that assumptions were violated, and the level of significance was 0.05.

## Results

### Sample

The final study sample comprised 38 AN patients and 48 HC participants. AN patients were selected based on the study inclusion and exclusion criteria and on the availability of all BID-CA data. Of the 50 AN patients treated during the study period, 12 (24%) were excluded. Of these, two (4%) performed only the T2 measurement, because they had been admitted before the measurements started, and nine (18%) performed only the T1 measurement, because they discontinued treatment prematurely (n = 5; 10%) or changed to another hospital or practitioner (n = 4; 8%). Finally, one AN patient (2%) was excluded due to a Turner syndrome diagnosis. There were no differences between included and excluded AN patients regarding basic clinical variables (age, BMI, duration of illness, prior inpatients treatments); however, excluded AN patients had a shorter treatment duration (cf. **Supplementary Tables 1** and **2**). In the HC group, 3 of the originally 51 participants were excluded due to enhanced psychopathology. In the final sample, structured clinical interviews and questionnaire data were available only for 32 of the included 38 AN patients. AN patients (M = 189.42 months, SD = 18.31) were younger^1^ than HC (M = 203,10 months, SD = 20.81), age: *t*(84) = −3.191, *p* = .002; had similar body height (AN: M = 1.68 m, SD = 0.06; HC: M = 1.68 m, SD = 0.05), *t*(84) = 0.113, *p* = .91; and, as expected, had lower BMI, *t*(80,65) = 12.19, *p* < .001, and higher psychopathology (**Table 1**). Most of the AN patients (N = 33; 86.8%) fully met diagnostic criteria, a majority had restrictive type AN (N = 27; 71.1%), and almost half of them (N = 18; 47.4%) had at least one comorbid disorder (**Table 2**). The average duration of illness before admission was merely 1 year (M = 11.63 months, SD = 10.47; cf. **Supplementary Table 1**). The majority of cases had their first inpatient treatment for AN (N = 27; 71.1%), but some also had one (N = 7; 18.4%) or two (N = 4; 10.5%) prior hospital stays. The average duration of inpatient treatment was approximately 5 months (*M* = 158.34 days, *SD* = 82.68), and the T2 measurement was performed between 30 days before and 98 days after discharge (M = 7.26 days, SD = 31.21). Deviations between discharge and T2 resulted from treatment extensions (N = 5) or measurements at the end of a subsequent day patient or outpatient treatment (N = 9). AN patients showed an average weight gain of nearly 11 kg (*M* = 10.97 kg, *SD* = 4.90). The average BMI_SDS_ at T2 (*M* = −0.66, SD = 0.66), corresponding approximately to the 24^th^ age percentile, and an average increase in BMI_SDS_ of about two standard deviation scores from T1 to T2 (**Table 3**) indicate significant and clinically relevant weight gain in the AN group.

**Table 1:** Psychopathology questionnaires.

	AN (n = 32)		HC (n = 48)			
	*M*	*SD*	*M*	*SD*	*d*	*p*
BDI-II	27.75	12.11	3.25	3.30	2.43	*p* < .001
SCARED	30.41	16.40	13.35	8.59	1.17	*p* < .001
BSQ	116.06	40.72	51.55	16.25	1.85	*p* < .001
EDI-C	227.97	61.76	97.35	33.78	2.37	*p* < .001
EDI-C DT	22.06	10.03	4.04	3.95	2.10	*p* < .001
EDI-C BD	30.00	9.55	10.21	7.46	2.15	*p* < .001

(BDI-II, Beck Depression Inventory 2; SCARED-D, Screen for Child Anxiety Related Disorders; BSQ, Body Shape Questionnaire; EDI-C, Eating Disorder Inventory for Children; DT, drive for thinness; BD, body dissatisfaction).

**Table 2:** Main and comorbid diagnoses.

Main diagnoses		Comorbid diagnoses	
Anorexia nervosa, restrictive type	27 (71.1%)	MDD	15 (39.5%)
Anorexia nervosa, binge-purge type	6 (15.8%)	Anxiety disorder	3 (7.9%)
Atypical anorexia nervosa (EDNOS)	5 (13.1%)	PTSD	1 (2.6%)
		OCD	1 (5.3%)
		Avoidant personality disorder	2 (2.6%)

(EDNOS, eating disorder not otherwise specified; MDD, major depressive disorder; PTSD, post-traumatic stress disorder; OCD, obsessive-compulsive disorder; multiple comorbid diagnoses per patient were counted separately).

**Table 3:** Body mass index.

	BMI		BMI_SDS_	
	*M*	*SD*	*M*	*SD*
HC	20.59	2.54	-0.29	0.84
AN T1	15.09	1.62	-2.83	1.28
AN T2	18.90	1.32	-0.66	0.66
AN T2–T1	3.80	1.74	2.17	1.18

(SDS, standard deviation scores).

### Body Image Distortion Indices

**Figure 1:**
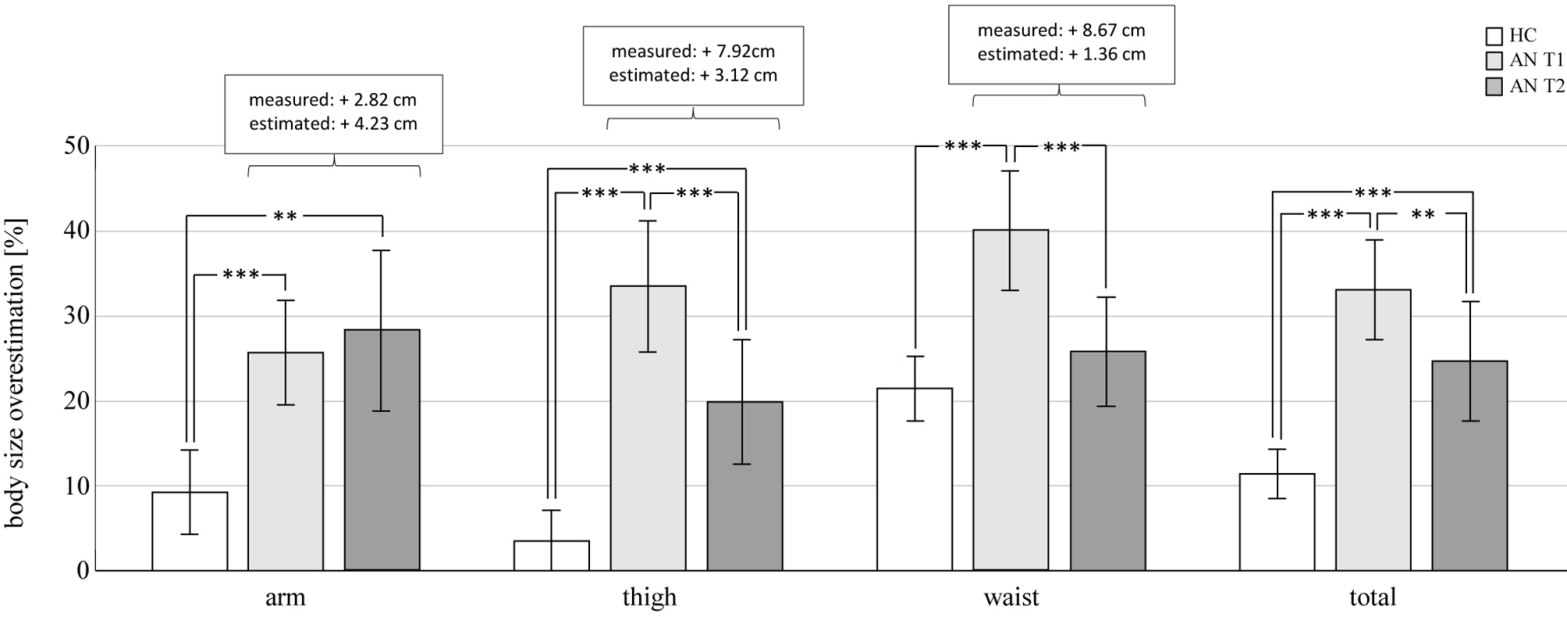
Body size overestimation. Body image distortion–indices (BID-I) − 100 indicate the percentage of overestimation. Increases in measured and estimated circumferences (cm) in AN patients from T1 to T2 are given in the white boxes. **p < .01; ***p < .001. HC, healthy control participants; AN, patients with anorexia nervosa.

As expected, at T1, AN patients exhibited larger BID-Indices compared to the HC group (**Table 4**; **Figure 1**). The respective ANOVA revealed an interaction of group × body part, *F*(2, 168) = 6.21, *p* = .002, η^2^ = .07, as well as a main effect of group, *F*(1, 84) = 50.79, *p* < .001, η^2^ = .38 (cf. **Figure 1**, **Table 3**). *Post hoc* *t*-tests (corrected for unequal variances when necessary) revealed that the expected effect of larger BID-Indices in the AN compared to the HC group was highly significant in all parts of the body—arm: *t*(84) = 4.24, *p* < .001; thigh: *t*(53.32) = 7.10, *p* < .001; and waist: *t*(58.01) = 4.69, *p* < .001 (cf. **Table 1**). The interaction arose (at least in part) through different effect sizes, with a medium effect at the arm (*r* = .42) and large effects at the thigh (*r* = .70) and waist (*r* = .52). As an additional control, one-way ANOVAs using measured body size as a covariate confirmed higher BID-Indices in AN patients *vs*. HC when controlling for a general effect of smaller body sizes—arm: F(1, 83) = 22.01, p < .001, η^2^ = .15; thigh: F(1, 83) = 18.93, p < .001, η^2^ = .19; and waist: F(1, 83) = 10.36, p = .002, η^2^ = 0.11. Moreover, BID-Indices differed by body part across groups, which was indicated by a main effect of body part, *F*(2, 168) = 25.617, *p* < .001, η^2^ = .24. *Post hoc t*-tests revealed that, across groups, BID-Indices were similar at the arm and thigh (*M* = 116.53, *SD* = 19.54 and *M* = 116.77, *SD* = 23.46, respectively), *t*(85) = 0.11, *p* = .910, *r* = .01, while overestimation was larger at the waist (*M* = 129.65, *SD* = 19.52) in comparison to the arm, *t*(85) = 6.26, *p* < .001, *r* = .56, and thigh, *t*(85) = 6.10, *p* < .001; *r* = .55.

**Table 4:** Body image distortion–indices (BID-I).

	Arm		Thigh		Waist		Total	
	*M*	*SD*	*M*	*SD*	*M*	*SD*	*M *	*SD*
HC	109.40	17.29	103.68	12.59	121.46	13.21	111.51	10.07
AN T1	125.70	18.71	133.48	23.50	140.02	21.45	133.07	17.82
AN T2	128.30	28.78	119.89	22.23	125.81	19.58	124.66	21.43

After treatment at T2, AN patients still exhibited larger BID-Indices compared to HC on some but not all body parts. The respective ANOVA revealed again a group × body part interaction, *F*(2, 168) = 7.97, *p* = .001, η^2^ = .09, and a main effect of group, but with a smaller effect size, *F*(1, 84) = 14.41, *p* < .001, η^2^ = .15. *Post hoc t*-tests now revealed larger BID-Indices in AN at the arm, *t*(57.11) = 3.60, *p* = .001, *r* = .43, and thigh, *t*(55.11) = 4.055, *p* < .001, *r* = .48, but not at the waist, *t*(61.66) = 1.18, *p* = .242, *r* = .15. Again, additional control analyses using one-way ANOVAs with measured body size as a covariate confirmed the results—arm: F(1, 83) = 14.42, p < .001, η^2^ = .15; thigh: F(1, 83) = 13.89, p < .001, η^2^ = .14; and waist: F(1, 83) = 1.28, p = .261, η^2^ = 0.02. Similar to T1, BID-Indices differed by body part across groups, indicated by a main effect of body part, *F*(2, 168) = 18.78, *p* < .001; η^2^ = .18). Across groups, BID-Indices were smallest at the thigh (*M* = 110.76, *SD* = 19.19), medium at the arm (*M* = 117.68, *SD* = 24.75), and largest at the waist (*M* = 123.37, *SD* = 16.32). *Post hoc t*-tests revealed significant differences in all comparisons, i.e., arm *vs*. thigh: *t*(85) = 3.50, *p* = .001, *r* = .36; waist *vs*. arm: *t*(85) = 2.56, *p* = .012, *r* = .27; waist *vs*. thigh: *t*(85) = 6.87, *p* < .001, *r* = .60.

Longitudinally, results confirmed the expected reduction of BID-Indices in AN patients after treatment but not in all parts of the body. The ANOVA comparing AN at T1 *vs*. T2 revealed an interaction of time × body part, *F*(2, 74) = 16.98, *p* < .001, η^2^ = .32), and main effects of body part, *F*(2, 74) = 3.51, *p* = .035, η^2^ = .09, and time, *F*(1, 37) = 8.82, *p* = .005, η^2^ = .19. *Post hoc t*-tests confirmed the expected reduction in BID-Indices from T1 to T2 at the thigh, *t*(37) = 4.51, *p* < .001, *r* = .60, and waist, *t*(37) = 3.93, *p* < .001, *r* = .54, but not at the arm, *t*(37) = −0.73, *p* = .468, *r* = .12. Moreover, BID-Indices differed by body part across both time points. Just like in the analyses at T1 across AN and HC groups, *post hoc t*-tests revealed that, across time points, AN patients’ BID-Indices were comparable at the arm (*M* = 126.99, *SD* = 21.68) and thigh (*M* = 126.68, *SD* = 20.90), *t*(37) = 0.12, *p* = .909, *r* = .02, while overestimation was larger at the waist (*M* = 132.92, *SD* = 17.24) compared to the arm, *t*(37) = 2.34, *p* = .025, *r* = .36, and thigh, *t*(37) = 2.29, *p* < .028, *r* = .35.^2^

### Relative Changes of Measured and Estimated Circumferences

Both measured and estimated circumferences increased longitudinally (**Table 5**; **Supplementary Figure 1**). Interestingly, reduced BID-Indices at T2 arose from a relatively lower growth of the estimated compared to the measured circumferences in some parts of the body. This was indicated by a method × body part interaction, *F*(2, 74) = 15.70, *p* < .001, η^2^ = .30, and a main effect of method, *F*(1, 37) = 7.34, *p* = .01, η^2^ = .17. The percentage changes of the measured and estimated circumferences were comparable at the arm, *t*(37) = −0.69, *p* = .493, *r* = .06, but they were larger for the measured *vs*. estimated circumference at the thigh, *t*(37) = 4.47, *p* < .001, *r* = .47, and waist, *t*(37) = 3.56, *p* = .001, *r* = .39. Moreover, a main effect of body part indicated that the percentage change differed by body part across methods, *F*(2, 74) = 7.31, *p* < .001, η^2^ = .17. In particular, the percentage change averaged across methods was larger at the waist than on the arm, *t*(37) = 3.80, *p* = .001, and thigh, *t*(37) = 2.72, *p* = .010, with no differences between the latter two, *t*(37) = 0.99, *p* = .327.

**Table 5:** Relative changes of measured and estimated circumferences (Changes from T1 to T2, AN group only).

	Measured				Estimated			
	cm		%		cm		%	
	*M*	*SD*	*M*	*SD*	*M*	*SD*	*M*	*SD*
Arm	2.82	1.59	12.29	7.46	4.23	5.76	14.46	19.77
Thigh	7.92	4.61	17.64	11.11	3.12	9.85	6.22	17.07
Waist	8.67	4.08	13.39	6.63	1.36	15.46	3.05	17.76

## Discussion

The present study investigated changes of the perceptive BID in adolescent AN patients after inpatient treatment. Treatment comprised a disorder-specific phased multimodal treatment plan including targeted body-psychotherapeutic work on both the perceptive and cognitive–affective body images. After treatment, AN patients achieved a significant weight gain. As a main result, this study shows that the distinct body size overestimation in AN patients observed at admission (T1) was clearly reduced after treatment (T2), though not on the arm but specifically at the thigh and waist (**Figure 1**). In fact, after treatment, AN patients’ BID-Indices at the waist no longer differed from HC. This indicates that perceptive BID in adolescent AN patients is modifiable and that, after treatment, AN patients were partly able to give a more realistic estimation of their body size.

We found that body size was overestimated by about 3–21% in HC participants and about 25–40% in underweight adolescent AN patients, and group differences were significant beyond effects that might be expected based on general smaller body sizes in the AN group (31). These findings are in line with previous findings using the BID-CA (24–26) and confirm the existence of a perceptive BID in AN (13). As the BID-CA is classified as a metric method of body size estimation, the relatively large effect sizes found in our study reflect the fact that metric methods yield in general larger effect sizes than depictive methods (13). Also, the observed reduction of this body size overestimation is in line with findings of reduced perceptive BID in AN patients after treatment (16, 19, 23). As prior studies used tasks involving size estimations of the whole body (visual size estimation, hoop task), it is an interesting new finding that, in this study, AN patients’ overestimation was improved only at the thigh and waist. Upon more closely inspecting the values underlying the calculation of BID-Indices, we found that weight regain led to a relatively even percentage increase of the measured circumferences in all body parts (12.3 to 17.6%, **Supplementary Figure 1**). However, while AN patients increased their estimations to a corresponding amount at the arm (14.5%), they only marginally changed their estimations on the thigh (6.2%) and waist (3.0%). Indeed, the significantly lower increase of estimated compared to measured circumferences at the thigh and waist apparently drove the observed reduction in the corresponding BID-Indices.

To interpret the results, a number of factors are worth considering. First, reduced BID-Indices at T2 might simply be a result of task repetition leading to training effects. However, the BID-CA is reported to have an acceptable test–retest reliability of 0.78 (25), and thus, it seems unlikely that task repetition can fully explain the results.

Second, AN patients might have deliberately reduced their estimations, even when they felt their body size was still larger, just because they became aware of their tendency to overestimate and tried to correct for it. Indeed, the results of the BID-CA were discussed with the patients, and the tendency to overestimate one’s own body size was extensively worked on during therapy. And although the patients were instructed to estimate circumferences as they felt at that moment, some patients reported an attempt to correct for the expected overestimation. If this was the case, however, it is remarkable that the patients still distinctly overestimated the size of their arm and thigh at T2. At least, despite their better knowledge, patients were not able to fully correct their tendency to overestimate.

Third, given that the estimations at the thigh and waist changed only marginally, the perceptive body image of AN patients might be considered as rather stable. Weight regain might have led the patients to grow into a preexisting perceptive body image, possibly still reflecting their body size prior to the weight loss. This idea fits with the observation that radical changes in the real constitution of the body, like limb amputation, may result in a situation where the perceptive body image is not updated, and individuals still feel a *phantom limb* (32). Such a phenomenon might be particularly prevalent in AN cases with rapid and severe weight loss. Indeed, both under- and overweight are associated with body size misperception (13, 33), suggesting that extreme weight changes might be associated with an impeded or delayed body image update. On the other hand, body size perception of a substantial proportion of overweight participants is accurate (33), and, hence, body image updates after weight gain are possible.

Finally, more realistic body size estimations and improved perceptive BID might be a result of therapeutic treatment. In particular, body-psychotherapeutic work on the perceptive component of body image, e.g., using exercises on multimodal integration, aimed to facilitate the conscious perception of the body and its boundaries. At the same time, the multimodal treatment plan also provided therapeutic work on the cognitive–affective and interpersonal component of the body image as well as on overall psychopathology. Both may have contributed to an improved body image and better task performances. Unfortunately, an analysis of the differential contributions of the diverse therapeutic treatments is not possible with the current study design.

Interestingly, although the first three points presumably all contributed to the observed results at least in part, they cannot fully explain the fact that changes of the BID-Indices are observed only at the thigh and waist. One possible explanation for this difference in body parts is that the thigh and waist, typically experienced as “problem zones” by the patients, received much more attention during (body-)psychotherapeutic treatment than the upper arm. Consequently, although this study was not designed as a clinical trial and the reasons for the observed changes may be diverse, these findings suggest that AN patients may benefit from therapeutic work targeting both cognitive–affective and perceptive aspects of BID.

Limitations of the present study concern, above all, the broad spectrum of possible factors influencing the observed BID-Index changes. To overcome this, future studies should control for test stability of the BID-CA, e.g., using a second test in HC and AN to compare changes from T1 to T2 in both groups. Moreover, the BID-CA in its original form could be better operationalized. For example, we included a short body perception exercise before the task to promote access to the implicit perceptive body image. The investigator pointed to the correct positions at each body part on her own body to improve understanding. In addition, we always measured the length of the string starting at the inner part of the end of the string touching the rest of the string, to avoid inaccuracies due to the strings’ thickness. Moreover, it is difficult to be sure that all participants perform the task in the same way, as some patients verbalized a conflict between their felt body size and their desire to correct for their known tendency to overestimate body size. A further development could thus be to use two separate task instructions to clarify the difference between the felt (perceptive) body size and the known (cognitive) body size, and to compare these two conditions. Furthermore, interpreting the observed perceptive BID changes in terms of a specific treatment effect would require a randomized controlled study using different treatment arms. For example, a group of AN patients with weight regain but without any targeted treatment of perceptive or cognitive–affective body image could be compared with groups of AN patients who received different add-on treatments specifically targeting perceptive and/or cognitive–affective BID. A further limitation of this study is the varying time point of the T2 measurement due to different treatment trajectories of individual patients. To avoid this, future studies could define a fixed time point, e.g., a fixed number of weeks after T1 (accepting less weight gain in some cases), or define a fixed target weight (accepting the exclusion of the group of patients who do not achieve it) for the T2 measurement. Finally, the study included only female participants and mainly rather severe inpatient cases, which limits the generalizability of the results.

The results of the present study show that the perceptive BID in adolescent AN patients is persistent but also modifiable. The BID-CA is a comprehensive and easy-to-use test that quantifies perceptive BID and helps to make AN patients aware of their tendency to overestimate their body size. Moreover, it enables treatment monitoring and repeated work on perceptive BID. Clinical experience shows that it may be quite challenging for AN patients to update their perceptive body image and that repeated therapeutic work on this is useful. More studies are needed on the perceptive component of BID to gain a better understanding of the factors that influence and facilitate change.

## Data Availability Statement

The datasets generated for this study are available on request to the corresponding author.

## Ethics Statement

The studies involving human participants were reviewed and approved by ethics committee of the Medical Association of Westphalian-Lippe and the University of Münster, Gartenstraße 210 - 214, 48147 Münster, Germany. Written informed consent to participate in this study was provided by the participants’ legal guardian/next of kin.

## Author Contributions

AD, IW and GR contributed to the conception and design of the study. AD and HF recruited the participants and collected the data. IW performed the statistical analysis and wrote the first draft of the manuscript. All authors contributed to the interpretation of the data and the manuscript revision, and read and approved the submitted version.

## Funding

Parts of the data included in this study were collected as part of a project funded by the German Research Foundation (project number: 409656150).

## Conflict of Interest

The authors declare that the research was conducted in the absence of any commercial or financial relationships that could be construed as a potential conflict of interest.

## Footnotes

1 As analyses using an age-matched HC sample (N = 35) revealed no qualitatively different results; the whole HC sample was kept.

2 An explorative correlation analysis of the relationship between BID-Index changes and weight gain revealed no significant result.

## Supplementary Material

Supplementary Figure 1 Relative changes of measured and estimated circumferences. Comparison of the percent change of measured and estimated circumferences in AN patients from T1 to T2. *** = p < .001.

Supplementary Table 1 Comparison of included and excluded AN patients. (BMI = Body Mass Index; SDS = Standard Deviation Scores; Duration of illness = months since begin of eating disorder symptoms estimated by AN patients and their parents at admission; Duration of treatment = Duration from admission to discharge from inpatient treatment).

Supplementary Table 2 Main and comorbid diagnoses of excluded AN patients. (EDNOS = Eating Disorder Not Otherwise Specified; MDD = Major Depressive Disorder; PTSD = Post Traumatic Stress Disorder; OCD = Obsessive-Compulsive Disorder; multiple comorbid diagnoses per patient were counted separately).
