# Supplementary figures and images for: Perceptive Body Image Distortion in Adolescent Anorexia Nervosa: Changes After Treatment
Source: Front Psychiatry. 2019 Oct 15;10:748. doi: 10.3389/fpsyt.2019.00748 (PMC6803517; doi:10.3389/fpsyt.2019.00748)

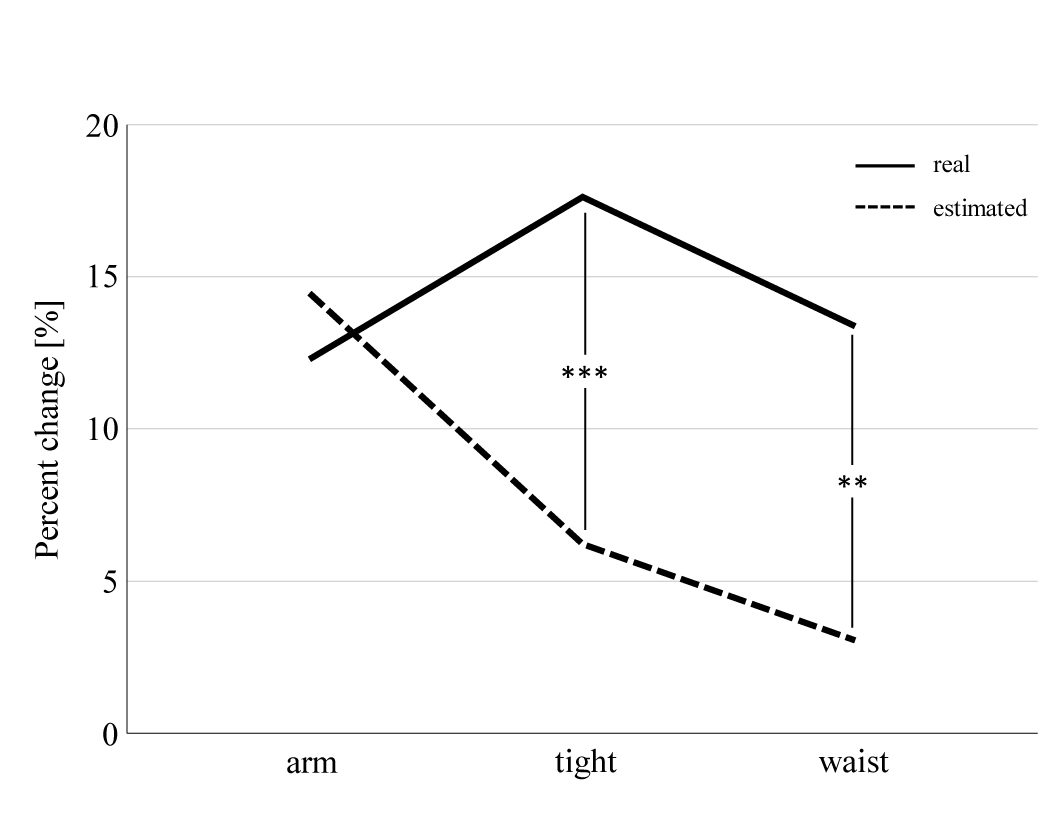

Supplement: Supplementary Figure 1 — Relative changes of measured and estimated circumferences. Comparison of the percent change of measured and estimated circumferences in AN patients from T1 to T2. *** = p < .001. [file Image_1.jpeg]
