# Supplementary material for: Perceptive Body Image Distortion in Adolescent Anorexia Nervosa: Changes After Treatment
Source: Front Psychiatry. 2019 Oct 15;10:748. doi: 10.3389/fpsyt.2019.00748 (PMC6803517; doi:10.3389/fpsyt.2019.00748)
Supplement: Supplementary Table 1 — Comparison of included and excluded AN patients. (BMI = Body Mass Index; SDS = Standard Deviation Scores; Duration of illness = months since begin of eating disorder symptoms estimated by AN patients and their parents at admission; Duration of treatment = Duration from admission to discharge from inpatient treatment). [file DataSheet_1.docx]

**Supplementary Material**

**Supplementary Table 1**

*Comparison of included and excluded AN patients.* (BMI = Body Mass Index; SDS = Standard Deviation Scores; Duration of illness = months since begin of eating disorder symptoms estimated by AN patients and their parents at admission; Duration of treatment = Duration from admission to discharge from inpatient treatment)

|  | AN included (n = 38) | | AN excluded (n = 12) | |  |  |
| --- | --- | --- | --- | --- | --- | --- |
|  | *M* | *SD* | *M* | *SD* | *d* | *p* |
| Age (months) | 189.42 | 18.32 | 187.08 | 24.04 | 0.12 | *p* = .723 |
| BMI | 15.09 | 1.62 | 15.42 | 1.92 | -0.20 | *p* = .558 |
| BMI_SDS_ | -2.83 | 1.28 | -2.61 | 1.50 | -0.16 | *p* = .625 |
| Duration of illness (months) | 11.63 | 10.47 | 13.91 | 3.60 | 0.20 | *p* = .176 |
| Prior inpatient treatments | 0.39 | 0.68 | 0.75 | 11.97 | 0.45 | *p* = .541 |
| Duration of treatment (days) | 158.34 | 82.68 | 95.50 | 87.70 | **0.75** | ***p* < .05** |

**Supplementary Table 2**

*Main and comorbid diagnoses of excluded AN patients.* (EDNOS = Eating Disorder Not Otherwise Specified; MDD = Major Depressive Disorder; PTSD = Post Traumatic Stress Disorder; OCD = Obsessive-Compulsive Disorder; multiple comorbid diagnoses per patient were counted separately)

| main diagnoses | | comorbid diagnoses | |
| --- | --- | --- | --- |
| Anorexia nervosa, restrictive type | 8 (66.7 %) | MDD | 4 (33.3 %) |
| Atypical Anorexia nervosa (EDNOS) | 4 (33.3 %) | Anxiety Disorder | 3 (25.0 %) |
|  |  | OCD | 2 (16.6 %) |
|  |  | Conduct Disorder | 2 (16.6 %) |
|  |  | Cannabis Use Disorder | 1 (8.3 %) |
